# Development of high affinity broadly reactive aptamers for spike protein of multiple SARS-CoV-2 variants†

**DOI:** 10.1039/d3ra01382k

**Published:** 2023-05-19

**Authors:** Thao T. Le, Donald J. Benton, Antoni G. Wrobel, Steven J. Gamblin

**Affiliations:** a Department of Chemistry, Imperial College London UK vtntle@gmail.com; b Structural Biology of Disease Processes Laboratory, The Francis Crick Institute UK Donald.Benton@crick.ac.uk

## Abstract

We have developed broadly reactive aptamers against multiple variants by alternating the target between spike proteins from different SARS-CoV-2 variants during the selection process. In this process we have developed aptamers which can recognise all variants, from the original wild-type ‘Wuhan’ strain to Omicron, with high affinity (*K*_d_ values in the pM range).

## Abstract 2

We have developed broadly reactive aptamers against multiple variants by alternating the target between spike proteins from different SARS-CoV-2 variants during the selection process.

## Graphical abstract

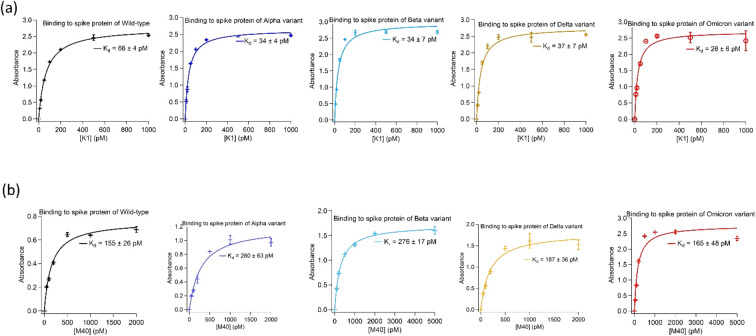

## Introduction

The SARS-CoV-2 virus infection is initiated by virus binding to the ACE2 cell-surface receptors, followed by fusion of the virus and cell membranes to release the virus genome into the cell.^1,2^ Both receptor binding and membrane fusion activities are mediated by the virus spike glycoprotein^3^ making it a prime candidate to be targeted for the development of vaccines as well as diagnostic and therapeutic reagents. There has been much focus on the development of both antibodies and aptamers against spike protein.^4,5^

Aptamers are single stranded DNA or RNA molecules that are selected *in vitro* to bind different types of targets including proteins and small molecules.^6–8^ They are often regarded as nucleic acid equivalence of antibodies.^9,10^ Aptamers can be advantageous over antibodies due to their capability to retain or regain correct three-dimensional structures for molecular recognition^11,12^ in physiochemical environments where the latter are often irreversibly damaged by denaturation.^13^ Thus, they have increasingly been using as alternatives for or partners of antibodies in diagnostic and therapeutic applications.^14,15^

The spike protein of SARS-CoV-2 is a homotrimeric protein. Each monomer is post translationally cleaved into S1 and S2 subunits. The S1 subunit contains two large domains, the N-terminal domain (NTD) and the Receptor Binding Domain (RBD) as well as two smaller subdomains. Both DNA and RNA aptamers have previously been selected against the spike protein or its subunits/domains to be used as diagnostic reagents or drug candidates.^16–23^

The rapid emergence of large numbers of new substitutions in SARS-CoV-2 spike protein^24^ poses a challenge in efficacy of vaccines, as well as the reagents used for diagnostics and therapeutics which are often specifically tailored for a particular variant. We aimed to develop aptamers that bind to spike protein of multiple different strains of SARS-CoV-2 virus, which improve their ability to bind future variants. To achieve this, we alternated the selection target between the spike protein from different variants of SARS-CoV-2 virus; the wild-type ‘Wuhan’ strain and the Alpha.

## Results and discussion

Two highly abundant aptamer sequences, K1 and M40, identified from the target-alternating selection showed exceptionally tight binding to spike protein of the wild-type and Alpha. Furthermore, when tested against spike protein from later emerged SARS-CoV-2 strains, *i.e.* Beta, Delta and Omicron, they displayed similar strong binding affinities.

**Fig. 1:**
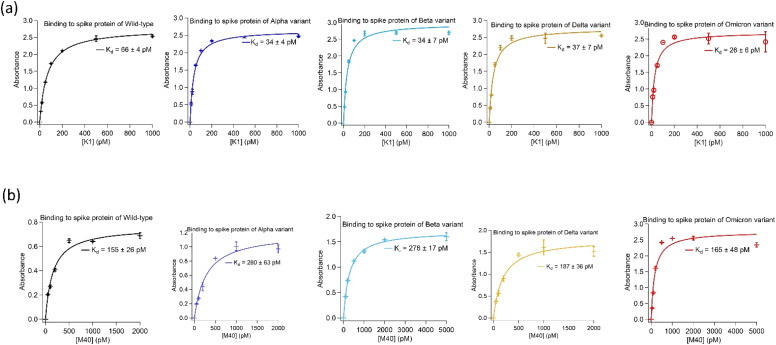
Binding affinities of the aptamers isolated using the target-alternating selection against spike protein of SARS-CoV-2 virus. (a) Binding of aptamer K1 to spike protein of wild-type, Alpha, Beta, Delta and Omicron. (b) Binding of aptamer M40 to spike protein of wild-type, Alpha, Beta, Delta and Omicron. Enzyme-linked oligonucleotide assays (ELONAs) were used to observe binding of the aptamers to spike protein. In the ELONAs, spike protein was immobilized on the microtiter plate wells and solutions of biotinylated aptamer in a range of concentrations were added. The aptamers had a biotin tag at their 3′end to facilitate enzyme (HRP) labelling through streptavidin conjugate (HRP-SA). The ELONAs were performed using buffer containing 20 mM Tris, 5 mM KCl, 2 mM MgCl_2_, 0.5 mM CaCl_2_ at pH 7.4. The *K*_d_ values obtained from fitting ELONA data to Langmuir isotherm on Igor software. Data for ELONAs were mean values obtained from two independent sets of samples performed and recorded parallelly. The error bars represent standard deviation of the values obtained from the two experiment sets. The aptamers also showed no significant changes in *K*_d_ values when replacing Tris by Hepes (20 mM Hepes, 5 mM KCl, 2 mM MgCl_2_, 0.5 mM CaCl_2_ at pH 7.4) in the assayed buffer. Binding data in Hepes buffer are presented in ESI, Fig. SI1.†

Both aptamers K1 and M40 were truncated to remove nucleotides in the 5′- and 3′-priming regions that showed no significant reduction in binding affinity to spike protein of SARS-CoV-2 virus in their absence. Binding affinities of truncated aptamers K1 (AATGGATCCA CATCTACGAG CTCGAACGCC CGGAGGAGAC GGGGGCAGGG CGTGTTCACT GCAGACTTGA CGAAGCT) and M40 (ATCTACGAAT TCTCCGCCAA GATAGAGGGT GGGCCGCGGA GAATTCACTGC) were determined using enzyme-linked oligonucleotide assays (ELONAs). Both aptamers showed high affinity toward all variants with dissociation constant (*K*_d_) values of K1 to spike protein were 26 ± 6 pM to 66 ± 4 pM whilst M40 were from 155 ± 26 pM to 280 ± 63 pM. In addition, binding affinity of both aptamers to spike protein of all variants were not affected when the Tris-based buffer (20 mM Tris, 5 mM KCl, 2 mM MgCl_2_, 0.5 mM CaCl_2_ at pH 7.4) was changed to Hepes-based buffer (20 mM Hepes, 5 mM KCl, 2 mM MgCl_2_, 0.5 mM CaCl_2_ at pH 7.4). ELONA binding curves of the truncated K1 and M40 to spike protein of the wild-type, Alpha, Beta, Delta and Omicron are shown in Fig. 1. The truncated sequences and the *K*_d_ values of aptamers K1 and M40 to spike protein of the strains are presented in Table 1.

**Table 1:** Sequences and *K*_d_ values of the aptamers, K1 and M40, binding to the spike protein of wild-type, Alpha, Beta, Delta and Omicron. ELONAs were performed using buffer containing 20 mM Tris, 5 mM KCl, 2 mM MgCl_2_, 0.5 mM CaCl_2_ at pH 7.4. The *K*_d_ values were obtained from ELONA data using to Langmuir binding isotherm on Igor software. Binding of the aptamers to spike protein also showed no significant changes in *K*_d_ values when replacing Tris by Hepes (20 mM Hepes, 5 mM KCl, 2 mM MgCl_2_, 0.5 mM CaCl_2_ at pH 7.4) in the assayed buffer as shown in ESI, Fig. SI1.

Aptamer	Sequence	*K*_d_ value (pM)				
		Wild-type	Alpha	Beta	Delta	Omicron
K1	5'-AATGGATCCACATCTACGA GCTCGAACGCCCGGAGGAGACGGGGGCAGGGCGTG TTCACTGCAGACTTGACGAAGCT-3'	66 ± 4	34 ± 4	34 ± 7	37 ± 7	26 ± 6
M40	5'-ATCTACGA ATTCTCCGCCAAGATAGAGGGTGGGCCGCGGAGAA TTCACTGC-3'	155 ± 26	280 ± 63	276 ± 17	187 ± 36	165 ± 48

**Fig. 2:**
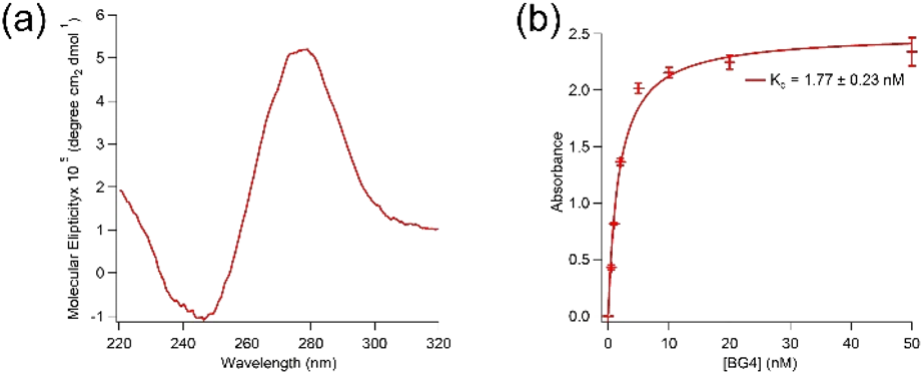
Puntative DNA G-quadruplex structures of aptamer K1 were characterised by CD and molecular recognition. (a) CD spectrum of 10 μM aptamer K1. (b) Binding of BG4, an anti G-quadruplex antibody, to aptamer K1 in complex with spike protein of wild-type CoV-2-SARS virus with a *K*_d_ value of 1.77 ± 0.23 nM, obtained from fitting ELONA data to Langmuir isotherm on Igor software. Data points were mean values obtained from two independent sets of samples. The error bars represent standard deviation of the values obtained from the two experiment sets. The CD measurements and ELONA were performed in buffer containing 20 mM Tris, 5 mM KCl, 2 mM MgCl_2_, 0.5 mM CaCl_2_ at pH 7.4.

Aptamer K1 was predicted to have one DNA G-quadruplex within its sequence with 23 possible combinations (overlaps) using QGRS Mapper.^25^ The overlaps are presented in Table SI1 in the ESI.†Fig. 2(a) shows a circular dichroism (CD) spectrum of aptamer K1 measured in the Tris buffer. It has been reported that DNA G-quadruplexes with the parallel topology display characteristic CD spectra of a dip at approximately 245 nm and a peak at around 265 nm whilst anti-parallel structures are expected to have a dip at about 265 nm and a peak at 295 nm.^26^ Aptamer K1 had a CD spectrum consisted of a dip at approximately 245 nm and a peak at around 280 nm, which is not fitted solely to the spectra of the parallel nor anti-parallel topology. However, the CD spectrum for aptamer K1 may constitute a mixture of different G-quadruplex conformations or a combination of both parallel and anti-parallel topologies.^27–29^ Putative G-quadruplex structures of aptamer K1 were further investigated using molecular recognition. It was reported that an antibody, BG4, bind DNA and RNA G-quadruplex structures in low nanomolar range.^30^ Aptamer K1 was recognised by BG4 in both free form (Fig. SI3†) or in complex (Fig. 2(b)) with spike protein. As shown in Fig. 2(b), BG4 binds aptamer K1 in complex with spike protein with *K*_d_ values of 1.77 ± 0.23 nM, which is similar to the *K*_d_ values previously reported for binding of BG4 to confirmed G-quadruplexes.^30^ This indicates that a G-quadruplex element is included in the three-dimensional structure of aptamer K1 in the complex formed with spike protein. It is worth noting that, this does not imply aptamer K1 retained its structure upon binding to the spike protein as confirmational changes may occur at the regions that do not involve in forming the G-quadruplex or at the G-quadruplex structure (rearrangement) or both.

**Fig. 3:**
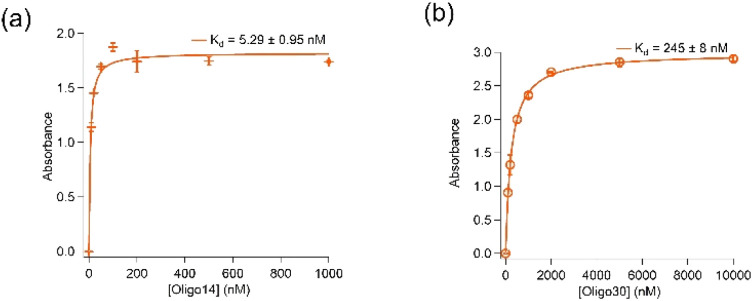
Contribution of nucleotides in binding of K1 and spike protein. (a) Oligo14 (14 nucleotides removed from both 5′- and 3′-ends) binds wild-type spike protein with a *K*_d_ value of 5.29 ± 0.95 nM. (b) Oligo30 (30 nucleotides removed from both 5′- and 3′-ends) binds wild-type spike protein with a *K*_d_ value of 245 ± 8 nM. ELONAs were performed in buffer containing 20 mM Tris, 5 mM KCl, 2 mM MgCl_2_, 0.5 mM CaCl_2_ at pH 7.4. The *K*_d_ values obtained from ELONA data used Langmuir model on Igor software.

Removal of further 14 (Oligo14) and 30 (Oligo30) nucleotides from both 5′- and 3′-ends resulted in significant loss in binding affinity of the aptamer to spike protein. As shown in Fig. 3, Oligo14 and Oligo30 bind spike protein of the wild type with *K*_d_ values of 5.29 ± 0.95 nM and 245 ± 8 nM, respectively, which are several orders of magnitude bigger than the *K*_d_ value obtained with aptamer K1. This confirms that these removed nucleotides from the 5′- and 3′- ends of aptamer K1 play a role in binding of the aptamer to spike protein. Oligo14 and Oligo30 however have their DNA G-quadruplex characteristics by CD and antibody recognition retained, indicating that the DNA G-quadruplex is only one binding element of the aptamer K1 and it needs these 30 nucleotides in order to achieve high affinity for spike protein. Sequences of Oligo14 and Oligo30 are presented in Table SI2 in the ESI.†

Most of the previously reported aptamers had relatively high affinity binding to the pure spike protein of SARS-CoV-2 virus with their *K*_d_ values mostly in low nanomolar range.^16–19,21,23^ However, it also showed that binding affinity of some of these aptamers were greatly enhanced (to picomolar range) by forming polymorphic complexes.^17,18^ In particular, a 'universal DNA aptamer' was recently reported that can bind spike protein of numerous variants with *K*_d_ values ranging from 2 to 10 nM.^16^ The aptamer was achieved by screening a library against wild-type spike protein for the first 13 rounds before finishing off by 1 round of selection for each of 5 variants. In our approach, we alternated two variants (wild-type and Alpha) from the start of the selection and kept the target rotating the whole selection process. Although the two approaches were different, they both resulted in aptamers that can bind spike proteins of multiple variants.

## Experimental

### Materials

Synthetic DNA libraries with a randomized region were obtained from Integrated DNA Technologies (IDT). Oligo nucleotides were made by Eurofins Scientific and IDT. Nitrocellulose membranes with 0.45 μm pore size and 13 mm diameter were from Merk Millipore. DNA polymerases (Pfu and Taq) were from New England Biolabs. dNTPs were from Promega. Streptavidin, HRP-streptavidin conjugate and TOPO™ TA Cloning sequencing kits were from Thermo Fisher Scientific. Other common chemicals were from Meck Millipore.

### Methods

#### Preparation of the spike protein

The spike protein of the wild-type and other variants of SARS-CoV-2 virus were prepared according to a previous publication.^2^

#### Selection of ssDNA aptamers for spike protein from multiple SARS-CoV-2 variants

A synthetic DNA library with sequence TAGGGAATGGAT CCACATCTACGA-(N)_35_-TTCACTGCAGAC TTGACGAAGCTT, where N_35_ is the randomized region with 35 nucleotides in length, was used for the selection. The reagents for synthesis of the 35 nucleotides in the random region were hand-mixed with a molar ratio of 30%, 30%, 20% and 20% for phosphoramidites of A, C, G and T, respectively. The starting synthetic DNA library used in this selection had 273 nmol (around 16 × 10^16^ molecules) and was used without purification to retain the diversity of sequence population. The library was suspended in 1 mL binding buffer containing 20 mM Hepes, 150 mM NaCl, 5 mM KCl, 2 mM MgCl_2_, 0.5 mM CaCl_2_, pH 7.4. It was then heated at 94 °C to denature folded structures formed in previous physicochemical conditions that may be different from the ones used for the selection. The solution was then left at room temperature for 20 minutes. An appropriate Spike stock solution containing 2.8 μg of the protein was subsequently added to the DNA solution and incubated at room temperature for 1 hour. The mixture was then filtered through a 0.45 μm pore size and 13 mm diameter nitrocellulose membrane and washed 4 times using 1 mL of the binding buffer each. The membrane-retaining sequences were recovered by 3 consecutive incubations in 270 μL of distilled water at 100 °C for 5 minutes to elute bound DNA sequences. The eluted aliquots were collected and then subjected to solvent extraction using Phenol : Chloroform : Isoamyl Alcohol (24 : 24 : 1 v/v) in order to remove the spike protein. The remaining eluted DNA sequences in the aqueous phase of the extraction were collected for PCR amplification in order to prepare a DNA enriched library for the next round of selection. For subsequent rounds, 10 nmol of the enriched libraries were used with 1.4 μg of the spike protein. The selection target was alternated between the spike protein from the wild-type and Alpha. Every 2 cycles, the enriched library was filtered through a nitrocellulose membrane before the enriched library-target binding to eliminate the membrane-binding sequences. Selection progress was monitored through binding of enriched libraries of rounds 2nd, 6th, 10th, 15th and 19th to the targets. The selection was concluded at round 20th as binding of round 19th enriched library to spike protein had reached saturation.

#### Amplification of the bound population and purification of the enriched libraries

The bound DNA sequences from the previous round of selection were amplified using asymmetric PCR with a mixture of *Pfu* and *Taq* polymerases in which 100 folds of concentrations of the forward primer to the reverse primer were used. After the amplification reaction, the solution was added with 1/10 volumes of 3 M sodium acetate and 2.5 volumes of absolute ethanol and incubated at −20 °C overnight (or −80 °C for 1 hour) for DNA precipitation. The solution was then centrifuged at 28,000×*g* for 1 hour and the precipitated DNA pellet was dried in an air-pump vacuum centrifugation and suspended in distilled water and purified using 10% TBE-Urea polyacrylamide gel electrophoresis.

#### Sequence identification and analysis

The DNA enriched library of the 20th rounds was amplified by symmetric PCR before inserted into pCR™^4^-TOPO™ TA vector (TOPO™ TA Cloning™ Kit of Thermo Fisher Scientific). The insert-carried plasmid population were transfected into One Shot™ TOP10 Chemically Competent *E. coli* cells. Individual *E. coli* colonies were picked, and their plasmids were purified and identified using Sanger sequencing. The sequencing data were analysed using Needle (EMBOSS) program of European Bioinformatics Institute (EBI).

#### Aptamer-spike protein binding affinity using enzyme-linked oligonucleotide assays (ELONAs)

Spike protein was immobilized on the microtiter plate (1 μg in 100 μL binding buffer/well). The plate was kept in a fridge overnight. The spike protein-immobilized wells were blocked with 1% of bovine serum albumin (BSA) and 2% of ovalbumin in binding buffer at room temperature for 1 hour. The wells then washed 4 times with the binding buffer supplemented with 0.05% Tween 20 (BBT) and 1 time with the binding buffer using 300 μL volumes. A 100 μL volume of each biotinylated aptamer concentration was added to a well and incubated at room temperature for 1 hour. The aptamers had a biotin tag at their 3′end to facilitate enzyme (HRP) labelling through streptavidin conjugate (HRP-SA). The wells were washed 3 times with the binding buffer supplemented with 0.05% Tween-20. Each drained well was added with 100 μL of streptavidin-HRP conjugate (21130, Thermo Fisher Scientific) diluted in binding buffer supplemented with 1% BSA and 2% ovalbumin The plate was incubated for another 40 minutes at room temperature. The wells were washed 2 times with BBT supplemented with 0.2% BSA and 0.4% ovalbumin and 1 time with binding buffer using 300 μL volumes before a 100 μL volume of TMB substrate solution (T0440, Merck) was added to each well. The reaction was quenched by adding a volume of 100 μL of 2 M H_2_SO_4_ solution and absorbance values at 450 nm was recorded. The absorbance values were plotted against aptamer concentrations using Langmuir binding model to obtain values for dissociation constants for aptamer-spike protein binding.

#### Aptamer truncation

Nucleotides were removed from the forward and reverse priming regions of the originally identified aptamer sequences. Binding of these shorten versions to the spike protein of wild-type SARS-CoV-2 virus were tested using ELONAs to find the truncated sequences. The shortest (truncated) sequences that do not have their binding affinities reduced can be used as the working sequences of the aptamers.

#### Sandwich enzyme-linked immunosorbent assays (Sandwich ELISAs)

The sandwich ELISAs were performed with the aptamer is sandwiched between spike protein and BG4 assayed in the binding buffer (20 mM Tris, 5 mM MgCl_2_, 1 mM CaCl_2_, 5 mM KCl, pH 7.4). Microtiter plate wells were first coated with spike protein as described above in ELONAs. 100 μL volumes of 1 μM solution of the biotinylated aptamer K1 were added to spike protein coated wells of the microtiter plate. The biotin tag is not needed but it was used in this assay due to availability of the biotinylated aptamer. The excess biotinylated aptamer was washed using 3 times of 300 μL of the binding buffer supplemented with 0.05% Tween 20. The wells with spike protein-aptamer were added 100 μL of FLAG tagged BG4 (Merck Millipore) solution. Detection was achieved with an HRP-conjugated anti-FLAG antibody and TMB substrate as described above in ELONAs.

#### Circular dichroism spectroscopy (CD)

10 mM of the aptamer solutions in the binding buffer (20 mM Tris, 5 mM MgCl_2_, 1 mM CaCl_2_, 5 mM KCl, pH 7.4) were heated to 94 °C for 5 minutes and let to cool at room temperature for 20 minutes before the CD measurements. The CD spectrum was obtained using a J715 spectropolarimeter (Jasco) with the buffer spectrum subtracted and zero correction at 320 nm.

## Conclusions

We developed aptamers that broadly bind spike protein from multiple variants of SARS-CoV-2 virus with high affinities by alternating targets during selection. The two identified aptamers, K1 (AATGGATCCA CATCTACGAG CTCGAACGCC CGGAGGAGAC GGGGGCAGGG CGTGTTCACT GCAGACTTGA CGAAGCT) and M40 (ATCTACGAAT TCTCCGCCAA GATAGAGGGT GGGCCGCGGA GAATTCACTGC), not only bind spike protein of wild-type and Alpha, their selection targets, but also Beta, Delta and Omicron, the later emerged strains with high affinity; *K*_d_ values of both aptamers to spike protein of variants were all in pM range. In addition, aptamer K1 showed characteristic of putative DNA G-quadruplexes. This selection approach may offer a tool to develop aptamers that can cope with emergence of new variants/strains that occurs frequently as the result of a pandemic's large number of infected hosts. The aptamers can be further investigated as candidates for diagnostic and therapeutic applications. In particular, aptamer-based lateral flow tests would be suitable for mass testing that is often needed during pandemics.

## Conflicts of interest

There are no conflicts to declare.

## Supplementary Material

RA-013-D3RA01382K-s001
