# Supplementary material for: Development of high affinity broadly reactive aptamers for spike protein of multiple SARS-CoV-2 variants
Source: RSC Adv. 2023 May 19;13(22):15322–6. doi: 10.1039/d3ra01382k (PMC10197177; doi:10.1039/d3ra01382k)
Supplement: RA-013-D3RA01382K-s001 [file RA-013-D3RA01382K-s001.pdf]

Electronic Supplementary Material (ESI) for RSC Advances.
This journal is © The Royal Society of Chemistry 2023

Supplementary Information

Development of High Affinity Broadly Reactive Aptamers for Spike Protein of Multiple SARS-CoV-2 Variants

Thao T. Le, Donald J. Benton, Antoni G. Wrobel and Steven J. Gamblin

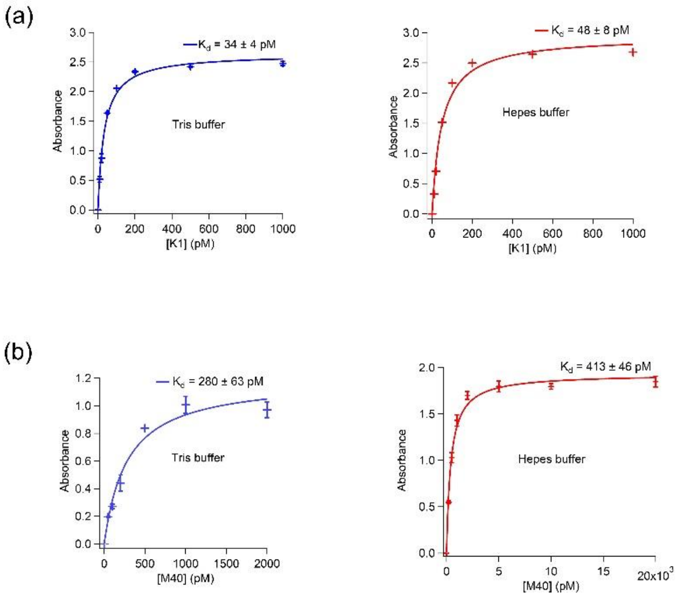

Figure SI1: (a) Binding of aptamer K1 in Tris buffer and Hepes buffer. (b) Binding of aptamer M40 in Tris buffer and Hepes buffer.

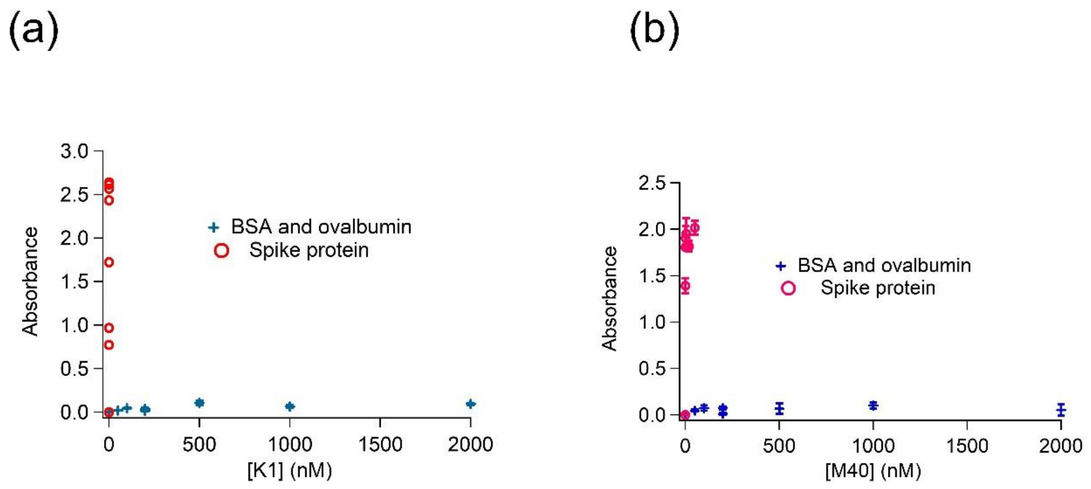

Figure SI2: Control experiments. (a) Binding of aptamer K1 to BSA and ovalbumin. (b) Binding of aptamer M40 to BSA and ovalbumin.

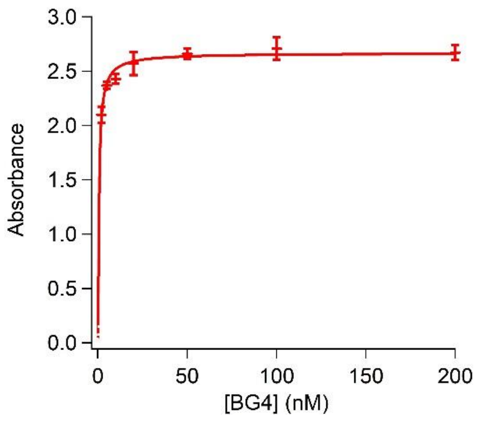

Figure SI3: Binding of BG4 to biotinylated aptamer K1 (immobilised on plate) by ELISA. Aptamer K1 had a biotin tag at its 3′-end to facilitate its immobilisation on the streptavidin-coated microtiter plate.

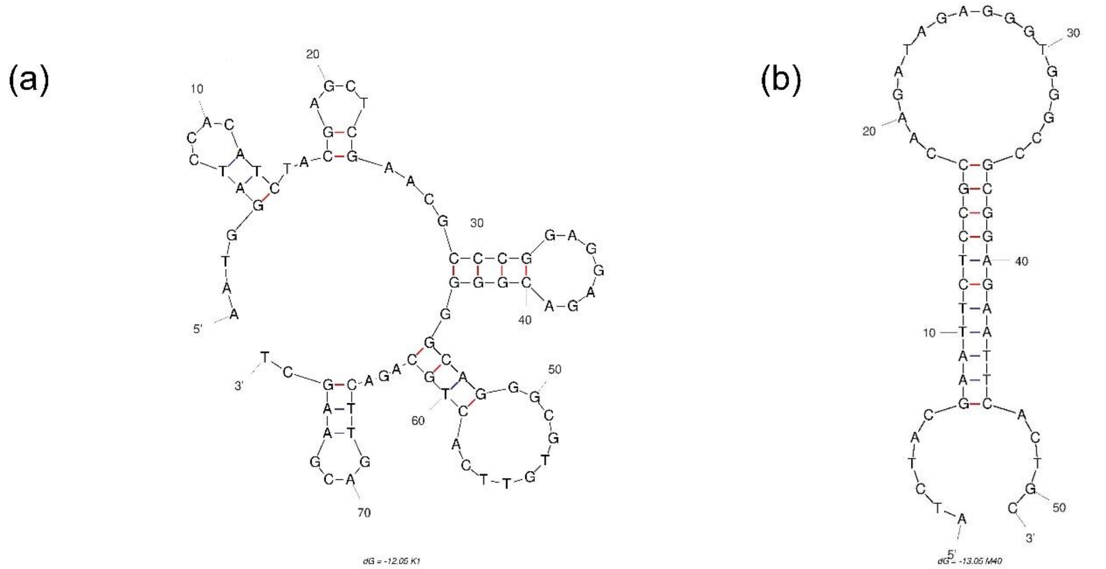

Figure SI4: Predicted secondary structures of the aptamers using MFold[1] at in solution having 150 mM Na^{+} and 5 mM Mg^{2+} and 25°C. (a) Predicted structure of aptamer K1. (b) Predicted structure of aptamer M40.

Table SI1: A predicted G-quadruplex of aptamer K1 with its overlaps by QSGR-Mapper[2]

Predicted structure Sample	Position	Length	QGRS	G-Score
P1	32	13	GGAGGAGACGGGG	17
P2	32	14	**GGAGGAGACGGGGG**	18
P3	32	14	GGAGGAGACGGGGG	16
P4	32	18	GGAGGAGACGGGGGCAGG	17
P5	32	18	GGAGGAGACGGGGGCAGG	17
P6	32	18	GGAGGAGACGGGGGCAGG	16
P7	32	18	GGAGGAGACGGGGGCAGG	15
P8	32	18	GGAGGAGACGGGGGCAGG	14
P9	32	18	GAGGAGACGGGGGCAGG	15
P10	32	18	GGAGGAGACGGGGGCAGG	13
P11	32	19	GGAGGAGACGGGGGCAGGG	16
P12	32	19	GGAGGAGACGGGGGCAGGG	17
P13	32	19	GGAGGAGACGGGGGCAGGG	16
P14	32	19	GGAGGAGACGGGGGCAGGG	15
P15	32	19	GGAGGAGACGGGGGCAGGG	14
P16	32	19	GGAGGAGACGGGGGCAGGG	15
P17	32	19	GGAGGAGACGGGGGCAGGG	13
P18	35	15	GGAGACGGGGGCAGG	17
P19	35	15	GGAGACGGGGGCAGG	18
P20	35	15	GGAGACGGGGGCAGG	16
P21	35	16	GGAGACGGGGGCAGGG	17
P22	35	16	GGAGACGGGGGCAGGG	18
P23	35	16	GAGACGGGGGCAGGG	16

Table SI2: Sequences of the truncated oligos

**Oligo14**	CCACATCTACGA GCTCGAACGCCCGGAGGAGACGGGGGCAGGGCGTG TTCACTGCAGACTTGA
**Oligo30**	ACGA GCTCGAACGCCCGGAGGAGACGGGGGCAGGGCGTG TTCACTGC

References

1 M. Zuker, Nucleic Acids Research, 2003. **31**(13): p. 3406-3415.
2 O. Kikin, L. D'Antonio, and P. S. Bagga, Nucleic Acids Research, 2006. **34**(suppl_2): p. W676-W682.
